# p53-independent mechanisms regulate the P2-*MDM2* promoter in adult astrocytic tumours

**DOI:** 10.1038/sj.bjc.6604643

**Published:** 2008-09-09

**Authors:** M Dimitriadi, G Poulogiannis, L Liu, L M Bäcklund, D M Pearson, K Ichimura, V P Collins

**Affiliations:** 1 Division of Molecular Histopathology, Department of Pathology, University of Cambridge, Level 3, Lab Block, Addenbrooke's Hospital, Box 231, Cambridge CB2 0QQ, UK; 2 Department of Oncology–Pathology, Karolinska University Hospital, Stockholm 171 76, Sweden

**Keywords:** *MDM2*, promoters, *TP53*, *p14* ^*ARF*^, SNP309, gliomas

## Abstract

The *MDM2* gene is amplified and/or overexpressed in about 10% of glioblastomas and constitutes one of a number of ways the p53 pathway is disrupted in these tumours. *MDM2* encodes a nuclear phosphoprotein that regulates several cell proteins by binding and/or ubiquitinating them, with p53 being a well-established partner. *MDM2* has two promoters, P1 and P2 that give rise to transcripts with distinct 5′ untranslated regions. Transcription from P2 is believed to be controlled by p53 and a single-nucleotide polymorphism (SNP309, T>G) in P2 is reported to be associated with increased risk for, and early development of, malignancies. The use of P1 and P2 has not been investigated in gliomas. We used RT–PCR to study P1- and P2-MDM2 transcript expression in astrocytic tumours, xenografts and cell lines with known *MDM2*, *TP53* and *p14*^*ARF*^ gene status. Both promoters were used in all genetic backgrounds including the use of the P2 promoter in *TP53* null cells, indicating a p53-independent induction of transcription. Transcripts from the P1 promoter formed a greater proportion of the total *MDM2* transcripts in tumours with *MDM*2 amplification, despite these tumours having two wild-type *TP53* alleles. Examination of SNP309 in glioblastoma patients showed a borderline association with survival but no apparent correlation with age at diagnosis nor with *TP53* and *p14*^*ARF*^ status of their tumours. Our findings also indicate that elevated MDM2 mRNA levels in tumours with *MDM2* amplification are preferentially driven by the P1 promoter and that the P2 promoter is not only regulated by p53 but also by other transcription factor(s).

The evolutionarily conserved *MDM2* gene (12q15) encodes a 491 amino-acid nuclear protein, whose activity and cellular localisation is believed to be controlled by post-translational modifications (Meek and Knippschild, 2003). For example, phosphorylation of MDM2 at Ser-166 and Ser-186 by the protein kinase Akt (also known as PKB) results in nuclear entry (Ashcroft *et al*, 2002). MDM2 is an E3 ubiquitin ligase, known to negatively regulate p53 by blocking its *trans*-activation domain and targeting it for ubiquitination and proteasome-mediated degradation (Momand *et al*, 1992; Haupt *et al*, 1997; Kubbutat *et al*, 1997). The tumour suppressor protein p14^ARF^ associates with and inhibits the E3 ubiquitin ligase activity of MDM2 permitting an accumulation of p53 and a consequent transcriptional response (Kamijo *et al*, 1998). MDM2 interacts with many other proteins including Rb1, E2F1, and the ribosomal proteins L5, L11 and L23, indicating that MDM2 is involved in a complex circuit of interactions, affecting among others the cell cycle and apoptosis (Zhang and Zhang, 2005). The details of the control of these interactions have still to be determined.

*MDM2* is amplified and/or overexpressed in a variety of human tumours of diverse tissue origins (Momand *et al*, 1998). Up to 10% of the most malignant astrocytic tumours, glioblastomas (WHO malignancy grade IV) (Louis *et al*, 2007) show *MDM2* gene amplification with consequent mRNA overexpression. This is generally associated with primary (*de novo*) glioblastomas that have wild-type *TP53* and *p14*^*ARF*^ alleles (Reifenberger *et al*, 1993; Ichimura *et al*, 2000). Consequently, amplification and/or overexpression of *MDM2* is believed to be an alternative mechanism for escaping p53-regulated control (Ichimura *et al*, 2000).

*MDM2* gene transcription is regulated by two promoters, P1 and P2. The P1 promoter is located upstream of exon 1 and is active at basal constitutive levels in most cells (Mendrysa and Perry, 2000). Although motifs of the P1 promoter important for its activity have been defined, its control is still not understood (Chang *et al*, 2004; Phillips *et al*, 2006). The second promoter (P2) is located in intron 1, it has two p53-responsive elements (Zauberman *et al*, 1995) and p53 is believed to initiate *MDM2* transcription from this promoter, thus forming an auto-regulatory feedback loop. Other p53-independent mechanisms have also been proposed (Qi *et al*, 1999; Ries *et al*, 2000; Phelps *et al*, 2003, 2005). In addition, a T>G polymorphism, referred to as SNP309 (rs2279744) in intron 1 of the human *MDM2* gene, has been suggested to affect P2 activity by increasing the binding affinity of the Sp1 transcription factor (Bond *et al*, 2004). This is thought to result in higher MDM2 protein levels, which would potentially attenuate the p53 pathway and might facilitate cancer formation (Bond *et al*, 2004).

Transcripts derived from the P1 promoter (P1-MDM2) do not utilise exon 2 and have exon 1 as their 5′ untranslated region (5′ UTR), whereas P2-derived transcripts (P2-MDM2) have exon 2 (Barak *et al*, 1994). Bases 5–7 of exon 3 form the start codon and are common to transcripts from both promoters. Translation of transcripts from the P1 promoter is eight-fold less efficient than translation of the P2 promoter transcripts (Landers *et al*, 1997), probably due to the presence of two short upstream open reading frames located in exon 1 (Jin *et al*, 2003).

The majority of studies have examined total MDM2 mRNA expression in normal and tumour tissues (Bueso-Ramos *et al*, 1995; Broll *et al*, 1999; Ko *et al*, 2000; Miyajima *et al*, 2001). The individual activity of the P1 and P2 promoters has been investigated in breast and oral cancer and head and neck squamous cell carcinoma (Ralhan *et al*, 2000; Millon *et al*, 2001; Okumura *et al*, 2002), but not in human gliomas. We have assessed how the gene status of *MDM2*, *TP53* and *p14*^*ARF*^ influences promoter usage in astrocytic gliomas (primary tumours, glioblastoma xenografts, glioblastoma cell lines). In addition, the SNP309 status was studied in glioblastoma patients and correlated to a number of genetic (i.e., *TP53* and *p14*^*ARF*^) and clinical (i.e., survival and age at diagnosis) parameters.

## Materials and methods

### Tumour tissue, xenografts and cell lines

A total of 73 gliomas including 56 glioblastomas (prefixed as GB), 10 anaplastic astrocytomas (prefixed as AA) and 7 astrocytomas (prefixed as A) were used in the study. In addition, xenografts from three glioblastomas and three glioma cell lines were studied. The primary tumours were classified according to WHO classification (Louis *et al*, 2007). Each tumour piece analysed had a minimum tumour cell content of 75% but generally greater than 90%, as assessed by histology. The tumours have been included in previous studies using the same identification numbers (Reifenberger *et al*, 1993; Ichimura *et al*, 2000, 2008; Liu *et al*, 2005). Xenografts hold the same number as the tumour from which they were derived with the suffix X followed by passage number. Collection and handling of tumour tissues and xenografts were as described (Schmidt *et al*, 1999; Ichimura *et al*, 2000). The characteristics of the cell lines Tp365MG and Tp265MG have been reported elsewhere (Collins, 1983). CCF-STTG1 was purchased from ATCC (Middlesex, UK). Supplementary Table 1 lists specimen numbers and their diagnosis, and indicates in which part of the study each specimen was used. Table 1 lists the *MDM2*, *TP53* and *p14*^*ARF*^ gene status of the tissues, xenografts and cell lines used. The study was approved by the Ethical Committee of the Karolinska Hospital (No. 91 : 16) and the Cambridge Local Research Ethics Committee, Cambridge, UK (ref. LREC 03/115).

### *TP53* analysis by multiplex PCR and *MDM2* SNP309 genotyping

DNA extraction from patients' peripheral blood and cell lines was as described previously (Ichimura *et al*, 1996). Multiplex PCR was performed by amplifying exon 2 (PC1046/PC1047) and exon 5 (PC929/PC931) of the *TP53* gene together with exon 35 (PC2419/PC2420) of an internal control gene (*DEPDC5*). The latter is known to be genetically normal in the samples investigated (Seng *et al*, 2005). Products were carried out on 2% agarose gels and visualised by ethidium bromide staining. For sequences of the primers used, see Supplementary Table 2. The *MDM2* SNP309 locus (rs2279744) was genotyped in the peripheral white blood cell DNA of 70 of the 73 astrocytic glioma patients in the series, using previously published primers and standard PCR conditions (Bond *et al*, 2004). The T to G variation at the 309th nucleotide of intron 1 of the *MDM2* gene was sequenced using an ABI PRISM 3100-Avant Genetic Analyser (Applied Biosystems, Warrington, UK) and Accelrys Gene 2.0 (Accelrys, Cambridge, UK) sequencing analysis software.

### RT–PCR of TP53 and P1- and P2-MDM2 transcripts

Total RNA was extracted from tumour pieces and cell lines as described (Ichimura *et al*, 1996). The generation of cDNA and the PCR conditions for all the experiments have been previously reported (Ichimura *et al*, 1996, 2008; Liu *et al*, 2005). For sequences of the primers used, see Supplementary Table 2. Primer pairs PC180/50 and PC446/50 were used to amplify exons 4–9 and exons 7–9 of the TP53 cDNA, respectively. To amplify the P1- and P2-MDM2 transcripts, forward primers PC3176 and PC3600 in the non-coding exons 1 or 2, respectively, were used with a common reverse primer PC3291 (exon 3) or PC3238 (exon 12) of the MDM2 cDNA. Standard cycling parameters (35 cycles) were used for the qualitative RT–PCR analysis on P1- and P2-MDM2 transcript expression. Products were carried out on 2% agarose gels and visualised by ethidium bromide staining. The real-time analysis of MDM2 mRNA was performed using a LightCycler^R^ in an identical manner to that described previously for a similar analysis of other genes (Ichimura *et al*, 2008), but using primer pairs PC3176/PC3291 and PC4570/PC4573 (see Supplementary Table 2) for the quantitation of P1- and P2-MDM2 transcript levels, respectively.

### Statistical analysis

For the statistical analysis, the raw data obtained from the quantitative RT–PCR analysis were transformed into log2 values. To assess the effect of *MDM2* gene status (amp or no amp) on P1- and P2-MDM2 mRNA levels, a Mann–Whitney *U* test was performed using glioblastomas with *MDM2* amplification, wt/wt *TP53* and wt/wt *p14*^*ARF*^ *vs* glioblastomas with no *MDM2* amplification, wt/wt *TP53* and wt/wt *p14*^*ARF*^. A Mann–Whitney *U* test was also used to compare the P1- and P2-MDM2 mRNA expression within different tumour grades (GBs *vs* AAs and As) that have no aberrations on *MDM2*, *TP53* and *p14*^*ARF*^ genes. A two-way ANOVA was used to test the effect of *TP53* and *p14*^*ARF*^ gene status or their combination on P1 and P2 transcript levels. For the latter test, tumours were separated into two categories: (i) those with wt/wt allelic status and (ii) those with at least one defective allele (i.e., wt/mut, wt/−, mut/mut, mut/− and −/−). Survival curves were obtained using the Kaplan–Meier method and statistical differences were analysed using the log-rank test. A Mann–Whitney *U* test was used to compare the age at diagnosis for glioblastomas in relation to the SNP309 genotype. Differences were accepted as significant for *P*<0.05. All statistical analyses were performed using Minitab 13.0 and SPSS 15.0 software packages.

## Results

### Transcription from the P2 promoter can be independent of p53 in astrocytic gliomas

**Figure 1:**
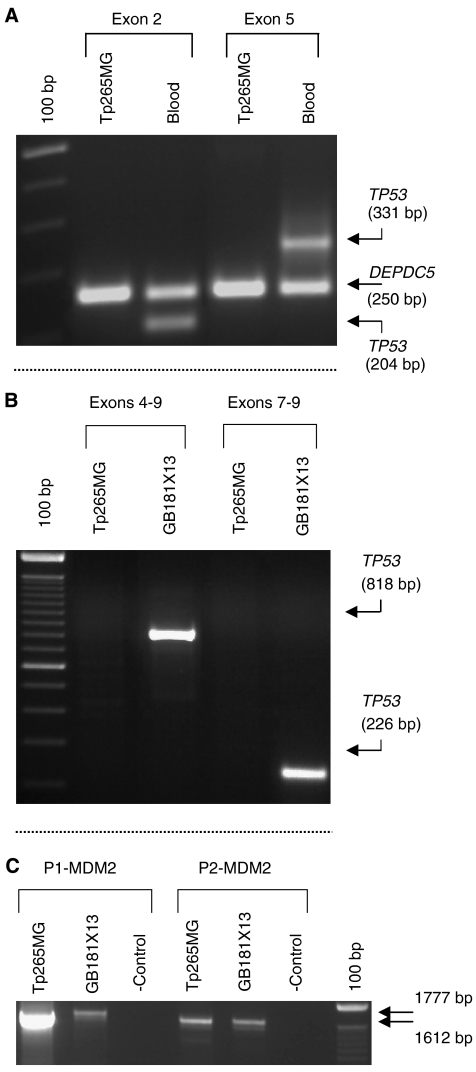
Agarose gels electrophoresis of PCR products confirming the homozygous deletion of *TP53* in Tp265MG and showing that expression from the P2 promoter is independent of *TP53* status. (**A**) Multiplex PCR using genomic DNA as template and independent pairs of primers (Supplementary Table 2) for exons 2 and 5 of *TP53,* and an unrelated retained locus (*DEPDC5*), confirming the homozygous deletion of *TP53* in Tp265MG. (**B**) RT–PCR analysis of TP53 transcripts encompassing exons 4–9 and exons 7–9 of the TP53 cDNA showing that no TP53 mRNA was detectable in Tp265MG. cDNA from the GB181X13 xenograft (*TP53*^wt/wt^) was used as a control template. (**C**) RT–PCR analysis of MDM2 transcripts using a forward primer located in either exon 1 (PC3176; 5′ P1 promoter) or exon 2 (PC3600; 5′ P2 promoter) with a common reverse primer (PC3238; sequence equivalent to 3′ UTR) and cDNA as template to amplify P1- and P2-MDM2 transcripts in Tp265MG. cDNA from GB181X13 (*TP53*^wt/wt^, expected to express MDM2 mRNA from both promoters) was used as a control. Note that MDM2 transcripts are expressed from both P1 and P2 promoters in Tp265MG glioma cell line and in GB181X13.

To investigate whether expression using the P2 promoter can occur in the absence of p53, we studied P1- and P2-MDM2 mRNA expression in a *TP53*-null glioma cell line (Tp265MG). Multiplex PCR and RT–PCR were first used respectively to confirm the *TP53* homozygous deletion and lack of endogenous TP53 mRNA in Tp265MG (Figure 1A and B). To identify the presence of P1- and P2-derived MDM2 transcripts in Tp265MG, primer sets that amplify the two distinct 5′ UTRs of MDM2 mRNA were used. Tp265MG was shown to express exon 2-derived transcripts (Figure 1C), and quantitative PCR confirmed the presence of P2 transcripts albeit at a very low level (Table 1).

In a preliminary study, the promoter usage of the *MDM2* gene was also investigated in a series of primary astrocytic tumours, comprising of 45 GBs, 5 AAs and 5 As (Supplementary Table 1) as well as in two additional glioblastoma cell lines (CCF-STTG1 and Tp365MG) and 3 glioblastoma xenografts (GB217X4, GB181X13 and GB166X1) with known *MDM2*, *TP53* and *p14*^*ARF*^ gene status using promoter-specific primers. Both P1- and P2-derived transcripts were detectable in all the samples including the tumours and xenografts that lack wild-type *TP53* (Supplementary Figure 1 and data not shown).

The majority of the primary tumours (67 of 73), the xenografts GB217X4 and GB166X1 and the cell line Tp265MG were examined by real-time PCR analysis confirming the P1- and P2-MDM2 usage (Table 1 and Supplementary Table 1). The P1/P2 ratio varied markedly from specimen to specimen, as might be expected due to the different known status of components of the p53 pathway and the content of normal cells in tumour specimens. The P1/P2 ratio was maximum in the Tp265MG cell line (Table 1). While it might be argued that in the tumour tissue samples analysed, all P2-derived transcripts originated from the normal cells (i.e., endothelial cells, pericytes, macrophages and so on) present, the P2 transcripts from the Tp256MG line and the GB166X1 xenograft (no wild-type *TP53* and use of human specific primers) could only have originated from the tumour cells. In addition, it is interesting that the transcripts in AA90 (homozygous deletion of *TP53*) show a P1/P2 ratio of 0.47. Overall, these findings provide evidence that p53 is not necessary for low-level P2-MDM2 transcription in astrocytic glioma cells.

### Variation in the usage of the P1 and P2 promoters in astrocytic gliomas

**Figure 2:**
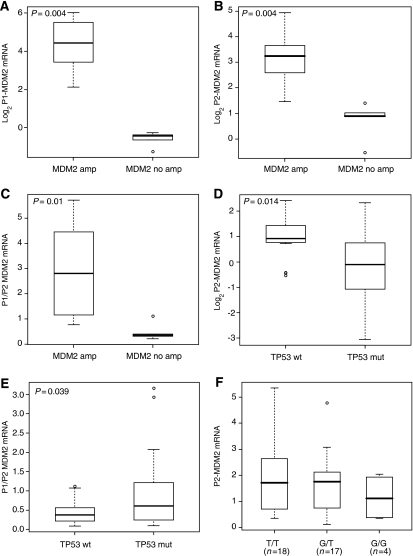
Box plot diagrams showing the distribution of the MDM2 mRNA levels (i.e., P1, P2 or P1/P2) obtained for glioblastomas in relation to their *MDM2* gene status (amplification or no amplification), *TP53* gene status (wild-type or mutation) and SNP309 genotype (T/T, G/T or G/G). The upper and lower limits of the boxes and the line across the boxes indicate the 75th, 25th percentiles and the median, respectively. The upper and lower horizontal bars indicate the 95th and 5th percentiles, respectively. Outliers are illustrated as circles. *P*-values are indicated at the top of the plots and were determined using the Mann–Whitney *U* test or the two-way ANOVA (see ‘Results’ section for details). (**A** and **B**) Log2 P1- and Log 2 P2-MDM2 mRNA expression in glioblastomas with and without *MDM2* gene amplification. (**C**) P1/P2 MDM2 mRNA ratio in glioblastomas with and without *MDM2* gene amplification. (**D**) Log2 P2-MDM2 mRNA expression in glioblastomas with wild-type or mutated *TP53*. (**E**) P1/P2 MDM2 mRNA ratio in glioblastomas with wild-type or mutated *TP53*. (**F**) P2-MDM2 mRNA expression levels in glioblastomas with no *MDM2* amplification in relation to their T/T, G/T or G/G genotypes for the SNP309 locus. Expression levels were measured as described in the ‘Materials and Methods’ section. There was no statistically significant difference in the expression levels between the T/T, G/T or G/G genotypes.

Tumours with *MDM2* amplification were grouped and compared to those with no *MDM2* amplification. This comparison showed a statistically significant increase in mRNA levels from both the P1 promoter (*P*=0.004, Mann–Whitney *U* test, Figure 2A) and the P2 promoter (*P*=0.004, Mann–Whitney *U* test, Figure 2B) in the *MDM2*-amplified group. This indicates that both promoters are utilised in cases with amplification of the *MDM2* gene. We next compared changes in the P1/P2 ratio in the same groups (Table 1). There was a significant difference in the P1/P2 ratio between tumours with *MDM2* amplification as compared to those without amplification (*P*=0.01, Mann–Whitney *U* test, Figure 2C), indicating that the elevated MDM2 mRNA expression seen in the *MDM2*-amplified astrocytic tumours is not equally driven by both promoters. *MDM2* amplification had a higher impact on P1 than P2 expression, as indicated from the comparison of the medians of (a) P1 expression in the amplified cases (median=22) *vs* P1 in the non-amplified cases (median=0.74) and (b) P2 expression in the amplified cases (median=9.46) *vs* P2 in the non-amplified cases (median=1.85).

The effect of *TP53* and *p14*^*ARF*^ gene status was then investigated to evaluate whether any *TP53* mutation (wt/mut, mut/mut or mut/−), *p14*^*ARF*^ hemi- and nullizygosity (wt/− or −/−) or a combination of the two have an effect on P1 and/or P2 transcript levels. A two-way ANOVA indicated that any *TP53* mutation was significantly associated with lower P2-MDM2 mRNA levels (*P*=0.014, two-way ANOVA, Figure 2D) and did not affect the activity of the P1 promoter (*P*=0.575, two-way ANOVA). This was also observed when changes in the P1/P2 ratio were examined with a correlation seen between *TP53* gene status and the P1/P2 ratio (*P*=0.039, two-way ANOVA, Figure 2E). Neither the *p14*^*ARF*^ gene status alone nor the combination of *TP53* and *p14*^*ARF*^ aberrations had any significant correlation with P1 or P2 transcript expression, nor with P1/P2 ratio (*P*>0.05, two-way ANOVA).

Finally, we tested whether there is a difference in the P1-, P2- or P1/P2 ratio of MDM2 mRNA among high-grade (GBs) *vs* lower-grade gliomas (AAs and As) with no *MDM2* gene amplification and wild-type *TP53* and *p14*^*ARF*^ genes. No statistical difference was observed (*P*>0.05, Mann–Whitney *U* test), suggesting that tumour grade does not affect MDM2 mRNA expression in a *TP53* and *p14*^*ARF*^ wild-type background.

### The P2 promoter polymorphism (SNP309) does not appear to be significant in astrocytic gliomas

To document the incidence of the SNP309 (rs2279744) polymorphism in astrocytic gliomas, DNA from peripheral white blood cells of 70 primary cases was studied (Supplementary Table 1). In addition to these, a glioblastoma cell line (Tp265MG) and a glioblastoma xenograft (GB181X13) were also investigated.

Table 1 summarises the genotypes obtained. Of the 70 patients genotyped for the SNP309, 34 (48.5%) were T/T, 27 (38.5%) were G/T and 9 (12.8%) were G/G. These frequencies are very similar to those seen in healthy Caucasian volunteers (48% T/T, 40% T/G and 12% G/G) (Bond *et al*, 2004). Notably, *TP53*-null specimens, Tp265MG (see above) and AA90 (confirmed by array-CGH, manuscript in preparation) were homozygous for the variant G allele.

To examine the effect of SNP309 on MDM2 expression, we compared the levels of P2-MDM2 mRNA in glioblastomas without *MDM2* gene amplification in relation to SNP309 genotype (Figure 2F). The results showed that glioblastomas homozygous for G/G did not have significantly higher levels of P2-MDM2 mRNA expression when compared to glioblastomas homozygous for T/T at this locus. Overall, the expression levels between the different SNP309 genotypes (T/T, G/T and G/G) did not appear to be substantially affected by the polymorphism at this locus.

As shown by others (Bond *et al*, 2004; Swinney *et al*, 2005) the age of cancer formation in patients with SNP309 (G/G) can vary greatly from those individuals with a T/T genotype. We compared the age distribution at the time of diagnosis for 54 glioblastoma patients who had homozygous T/T genotype with that of patients who had either heterozygous (G/T) or homozygous (G/G) variant genotypes at SNP309 (Table 1). The median age at diagnosis was identical for T/T genotype and G/T or G/G genotype (61 years). There was no association between T and G carriers in relation to age at diagnosis in glioblastomas (*P*=0.658, Mann–Whitney *U* test).

**Figure 3:**
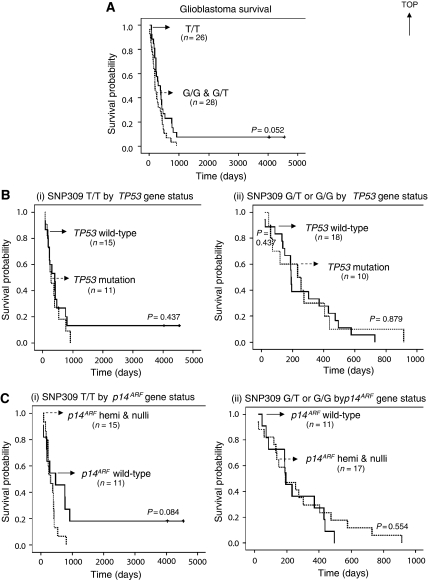
SNP309 in relation to glioma tumourigenesis. (**A**) Survival curves of patients with glioblastoma, comparing those with T/T to those with G/G or G/T for MDM2 SNP309. A borderline association in survival based on the presence or absence of the G allele was observed. (**B**) MDM2 SNP309 genotype in glioblastomas and *TP53* status (i.e., wild-type or mutation) in relation to survival. (i) Survival curves of glioblastoma patients with the common SNP309 genotype (T/T) by *TP53* gene status. (ii) Survival curves of glioblastoma patients with the variant SNP309 genotype (G/T or G/G) by *TP53* gene status. (**C**) MDM2 SNP309 genotype of glioblastomas and *p14*^*ARF*^ gene status (i.e., wild-type or hemi/nullizygosity) in relation to survival. (i) Survival curves of glioblastoma patients with the common SNP309 genotype (T/T) by *p14*^*ARF*^ gene status. (ii) Survival curves of glioblastoma patients with the variant SNP309 genotype (G/T or G/G) by *p14*^*ARF*^ gene status. The crosses indicate censored patients. Note that no significant difference was observed in survival in conjunction with *TP53* or *p14*^*ARF*^ gene status.

A Kaplan–Meier analysis was performed to test if the occurrence of this gene alteration had an effect on the prognosis of the glioblastoma patients. Patients without the polymorphism (T/T) showed a median survival of 337 days, whereas patients carrying a G allele (G/T or G/G) had a median survival of 193 days. Even though the median survival values appear quite different, only a borderline statistical association (*P*=0.052) in the Kaplan–Meier curves from glioblastomas with T/T compared with a G/G or G/T genotype could be shown in the relatively small group (*n*=54) (Figure 3A). In addition, the effect on survival of G/G (as compared to G/G+G/T) against T/T in glioblastoma patients was also tested and no significant association was observed (*P*=0.341, log-rank test). Although it has been suggested that the oestrogen signalling pathway allows the G allele to accelerate tumour formation in women (Bond and Levine, 2007), while our data did not include any G/G females, a preliminary comparison of the G/T against T/T female patients with glioblastoma did not show any association with survival (*P*=0.748, log-rank test).

A prognostic value of *TP53* gene status in relation to SNP309 genotype has not been demonstrated in glioblastomas. Therefore, we investigated whether SNP309 status might interact with differing *TP53* status to modify the survival of glioblastoma patients. Of the 54 glioblastomas analysed, 35 (65%) had wild-type *TP53* (wt/wt or wt/−) and 19 (35%) had at least one mutated *TP53* allele (wt/mut, mut/− or mut/mut). As shown by Kaplan–Meier analyses, SNP309 status did not affect the association between *TP53* gene status and survival in glioblastomas (Figure 3B).

We also examined the impact of *p14*^*ARF*^ gene status, as it is one of the central nodes in the p53 pathway. In the glioblastoma series studied, 22 of 54 (41%) had wild-type *p14*^*ARF*^ alleles, whereas 32 of 54 (59%) had lost at least one wild-type *p14*^*ARF*^ allele. As shown in Figure 3C, SNP309 status did not act synergistically with *p14*^*ARF*^ gene status to significantly alter survival in glioblastomas.

## Discussion

The usage of the two promoters of the *MDM2* gene was analysed here for the first time in astrocytic tumours, glioblastoma xenografts and glioblastoma cell lines with known *MDM2*, *TP53* and *p14*^*ARF*^ gene status. By documenting P1- and P2-MDM2-induced transcripts in a glioblastoma cell line (Tp265MG) with no *MDM2* gene amplification and homozygously deleted *TP53* and *p14*^*ARF*^, it was demonstrated that exon 2-derived transcripts, that is, transcripts initiated from the supposedly p53-responsive promoter (P2), were detectable even in the complete absence of all protein products of the *TP53* gene. Similar findings were made in an anaplastic astrocytoma case (AA90) with no *MDM2* gene amplification, homozygously deleted *TP53* and two wild-type copies of *p14*^*ARF*^ (Table 1). In addition, GB103 retains only one allele of *TP53* with the R175H mutation (Backlund *et al*, 2003) and the tumour also expresses MDM2 transcripts from the P2 promoter. The R175H mutation would affect all known splice variants of *TP53* (Bourdon *et al*, 2005) and has been shown to be transactivation defective (Soussi and Wiman, 2007).

P1- and P2-induced transcripts were expressed from other astrocytic gliomas of the series, which also lacked wild-type *TP53.* There were seven glioblastoma cases (GB164, GB61, GB131, GB29, GB166, GB16 and GB33) that showed loss of one *TP53* allele and mutation of the DNA-binding domain in the retained allele (Ichimura *et al*, 2000; Backlund *et al*, 2003). Such mutations affect all the proteins encoded by *TP53* transcript variants (Bourdon *et al*, 2005). While there is no information about the impact on function of the specific mutations affecting each case, these seven tumours can only generate mutant p53 tetramers, which according to the model proposed by Chan *et al* (2004) will be inactive. Finally, GB132 retained only one mutated *TP53* copy (R342X), affecting the sequence coding for the p53 oligomerisation domain, and this would prevent the formation of full-length p53 tetramers and the oligomerisation of the six other isoforms known to utilise this region of code (i.e., p53*β*, p53*γ*, Δ40p53*β*, Δ40p53*γ*, Δ133p53*β* and Δ133p53*γ*), but would not impact on two p53 isoforms (i.e., Δ40p53 and Δ133p53) (Ichimura *et al*, 2000; Backlund *et al*, 2003; Bourdon *et al*, 2005).

While it can be argued that the P2-derived MDM2 transcripts came from normal cells present in the tumour tissue analysed, both the Tp265MG cell line and the GB166X1 xenograft contain no normal human cells, yet both expressed the P2 transcripts (primers used for analysis of the xenografts were human-sequence specific) and this would argue for at least some of the P2 transcripts coming from the tumour cells themselves. Overall, our findings provide evidence that (i) wild-type p53 is not necessary to induce MDM2 transcription through the p53-responsive promoter (P2) and that (ii) other transcriptional factor(s) can regulate MDM2 mRNA expression through P2 in astrocytic gliomas that lack endogenous p53 protein expression.

Studies have shown that different p73 isoforms can transactivate the P2-*MDM2* promoter in p53-null cells (Zhu *et al*, 1998; Alarcon-Vargas *et al*, 2000; Wang *et al*, 2001), although this is debated (Yu *et al*, 1999). The *TP73* gene is located at 1p36, a region reported to show deletions in astrocytic gliomas (Barbashina *et al*, 2005). However, this series of cases has been studied in detail as to their copy-number status at 1p36 and this has shown that the majority of the tumours have two copies of *TP73* (Ichimura *et al*, 2008). The 13 cases that had only one copy of *TP73* (A50, A23, A30, AA34, AA49, GB96, GB250, GB3, GB52, GB56, GB63, GB84 and GB41) retained two wild-type copies of *TP53* (Ichimura *et al*, 2008). Thus, the p53-independent expression of P2-*MDM2* could be explained by p73 in astrocytic gliomas. Less is known about the *TP63* gene, but the p63α isoform has also been shown to weakly activate the MDM2 promoter in H1299, a p53-null non-small cell lung carcinoma cell line (Dohn *et al*, 2001).

Other functional, p53-independent elements have been reported in the P2 promoter. These include two thyroid hormone response elements (Qi *et al*, 1999) and an AP1-ETS motif together with a non-conserved upstream repeat sequence (nnGGGGC)_5_ (Phelps *et al*, 2003). At present, there are no data to indicate the relevance of p53-independent *cis*-acting elements for P2-*MDM2* promoter activity in gliomas with no wild-type *TP53*.

In an attempt to provide further insights into the contribution of P1 and P2 promoters on the regulation of MDM2 mRNA expression in astrocytic gliomas, quantitative RT–PCR analysis was used. Amplification of the *MDM2* gene in glioblastomas with wild-type *TP53* and *p14*^*ARF*^ alleles was clearly reflected at the transcriptional level. Although expression from both promoters was increased in cases with *MDM2* amplification, the amplification event had a higher impact on the P1 promoter. Assuming that the MDM2/p53 autoregulatory negative feedback loop is functional at the protein level in astrocytic tumours with amplified copies of the *MDM2* gene, one would expect the P1 promoter to give rise to high MDM2 mRNA and protein levels in these neoplasms, as all *MDM2* gene coded proteins with a p53-binding domain would inhibit the transcriptional activation of the P2 promoter by p53, reducing MDM2 mRNA expression from this promoter. This would result in higher MDM2 mRNA levels from the P1 promoter, as was observed.

Importantly, there also appeared to be a clear correlation between P2-MDM2 transcript levels and *TP53* gene status (Figure 2D). The statistical analyses of these data are in agreement with two independent studies in oral squamous cell carcinomas and in cell lines established from head and neck cancers or sarcomas, where P2-MDM2 transcript levels correlated with *TP53* gene status (Ralhan *et al*, 2000; Millon *et al*, 2001). In contrast, very little correlation was observed between P1/P2 values and p53 mutational status in human breast cancer specimens (Okumura *et al*, 2002).

Overall, it would seem that complete loss of wild-type p53 is not sufficient to inhibit the P2 promoter in astrocytic gliomas. Thus, P2 transcripts cannot be used to predict the presence of transcriptionally active p53 in astrocytic gliomas as has been suggested in other tumours by some authors (Bull *et al*, 1998; Millon *et al*, 2001).

In an attempt to further understand control of the P2 promoter in these tumours, we examined the SNP309 (rs2279744). This polymorphism in the P2 promoter has been found to affect the binding of the Sp1 transcription factor to the promoter sequence and the G allele has been associated with increased binding of Sp1 and expression of MDM2 transcripts and protein. The G allele has also been associated with accelerated tumour formation in both hereditary and sporadic cancers in humans (Bond *et al*, 2004). Sequencing of the SNP309 region of the P2 promoter in the peripheral blood white cell DNA of 70 astrocytic glioma patients showed that 48% of the patients were homozygous for T/T, 38% heterozygous for T/G and 12% homozygous for G/G. These frequencies were similar to the small cohort of healthy Caucasian volunteers studied by Bond *et al* (2004). Interestingly, both Tp265 MG and AA90 with homozygous deletion of *TP53* were homozygous for the G allele. The T to G substitution, which increases the binding affinity of the Sp1 transcriptional activator, might facilitate MDM2 mRNA expression from the P2 promoter in these samples. However, the P2-MDM2 expression observed in astrocytic gliomas with no wild-type *TP53* cannot be solely explained by the involvement of the Sp1 *trans*-acting factor, as 5 of 9 (55.5%) of these cases were found to be homozygous for the T allele (Table 1).

The G/G and G/T SNP309 in patients with glioblastoma showed a borderline association with poorer survival but no correlation with age at diagnosis or with the *TP53* and *p14*^*ARF*^ status of their tumours. Our data are in agreement with three independent studies, where SNP309 was shown not to have a significant involvement in glioma tumourigenesis (El Hallani *et al*, 2007; Tsuiki *et al*, 2007; Idbaih *et al*, 2008). Null results have also been reported in other types of cancer in relation to this polymorphism (Campbell *et al*, 2006; Petenkaya *et al*, 2006; Pine *et al*, 2006; Talseth *et al*, 2007).

Finally, to examine the effect of the SNP309 polymorphism on *MDM2* expression, the MDM2 mRNA transcriptional levels obtained by quantitative RT–PCR analysis were compared with SNP309 genetic status. In contrast to previous reports (Bond *et al*, 2004; Hong *et al*, 2005; Sanchez-Carbayo *et al*, 2007), the data did not show that G/G patients have a significantly higher P2-MDM2 mRNA expression levels as compared with the levels seen in astrocytic glioma patients with the T/T genotype. Overall, larger, prospective studies are needed to verify whether there is a clear involvement of this or other *MDM2* polymorphism(s) in glioma tumourigenesis.

In summary, we report that both the P1 and P2 promoters are used in all genetic backgrounds, including the use of the P2 promoter in *TP53*-null cells, indicating a p53-independent induction of transcription from P2. In glioblastomas with amplification of the *MDM2* gene, transcripts from the P1 promoter dominate despite all such cases having two wild-type *TP53* alleles. We also found no clear correlation between the SNP309 (rs2279744) locus in *MDM2* and age of presentation or survival in glioblastoma patients.

## Figures and Tables

**Table 1:** Gene status of *MDM2*, *TP53* and *p14^ARF^*, P1- and P2-MDM2 transcript levels of each specimen and SNP309 genotype and age at diagnosis of the corresponding patient

**Specimen no.**	***MDM2***	***TP53***	***p14^ARF^***	**P1^a^**	**P2^a^**	**P1/P2**	**SNP309**	**Age at diagnosis**
A22	No amp	wt/wt	wt/wt	0.84	1.01	0.832	G/G	NA
A54	No amp	wt/wt	wt/wt	0.68	2.02	0.337	T/T	NA
A25	No amp	wt/wt	wt/wt	0.42	0.52	0.808	T/T	NA
A50	No amp	wt/wt	wt/wt	0.40	0.63	0.635	G/T	NA
A7	No amp	wt/wt	wt/wt	0.07	0.49	0.143	G/T	NA
A23	No amp	wt/wt	wt/wt	0.58	0.63	0.921	T/T	NA
A30	No amp	wt/wt	wt/wt	0.30	0.24	1.250	T/T	NA
AA104	No amp	wt/wt	wt/wt	1.77	1.05	1.686	G/T	NA
AA34	No amp	wt/wt	wt/wt	2.27	1.02	2.225	T/T	NA
AA59	No amp	wt/wt	wt/wt	0.07	0.27	0.259	T/T	NA
AA76	No amp	wt/wt	wt/wt	0.80	3.42	0.234	G/T	NA
AA107	No amp	wt/wt	wt/wt	0.33	1.89	0.175	G/T	NA
AA110	No amp	wt/wt	wt/wt	6.44	14.07	0.458	G/T	NA
AA15	No amp	wt/wt	wt/wt	0.36	0.73	0.493	T/T	NA
AA50	No amp	wt/wt	wt/wt	0.36	0.44	0.818	T/T	NA
AA49	No amp	wt/wt	−/−	NA	NA	NA	NA	NA
AA90	No amp	−/−	wt/wt	0.11	0.230	0.478	G/G	NA
GB180	Amp	wt/wt	wt/wt	25.72	30.9	0.832	G/G	42
GB217	Amp	wt/wt	wt/wt	12.60	2.77	4.549	G/G	63
GB267	Amp	wt/wt	wt/wt	4.37	5.73	0.763	T/T	63
GB245	Amp	wt/wt	wt/wt	9.30	6.34	1.467	T/T	61
GB246	Amp	wt/wt	wt/wt	18.28	9.4	1.945	G/T	62
GB35	Amp	wt/wt	wt/wt	41.60	9.52	4.370	G/T	71
GB37	Amp	wt/wt	wt/wt	50.68	13.86	3.657	T/T	40
GB90	Amp	wt/wt	wt/wt	65.46	11.44	5.722	T/T	61
GB140	Amp	wt/wt	wt/−	9.91	5.66	1.751	G/T	42
GB223	Amp	wt/wt	wt/−	4.02	8.26	0.487	T/T	65
GB7	Amp	wt/wt	wt/−	129.97	12.06	10.777	T/T	41
GB81	Amp	wt/wt	wt/−	23.76	12.95	1.835	T/T	65
GB237	Amp	wt/−	wt/wt	NA	NA	NA	G/T	73
GB96	No amp	wt/wt	wt/wt	0.42	2.04	0.206	G/G	51
GB75	No amp	wt/wt	wt/wt	0.77	0.69	1.116	T/T	31
GB149	No amp	wt/wt	wt/wt	0.74	1.84	0.402	G/T	61
GB247	No amp	wt/wt	wt/wt	0.64	1.85	0.346	G/T	34
GB250	No amp	wt/wt	wt/wt	0.83	2.64	0.314	T/T	12
GB30	No amp	wt/wt	wt/−	NA	NA	NA	G/G	37
GB144	No amp	wt/wt	−/−	1.20	5.36	0.224	T/T	58
GB24	No amp	wt/wt	−/−	0.14	1.64	0.085	G/T	61
GB18	No amp	wt/wt	−/−	0.55	0.74	0.743	G/T	51
GB3	No amp	wt/wt	−/−	2.66	3.72	0.715	T/T	67
GB32	No amp	wt/wt	−/−	2.09	1.95	1.072	T/T	47
GB34	No amp	wt/wt	−/−	0.45	1.77	0.254	T/T	72
GB52	No amp	wt/wt	−/−	0.14	1.65	0.085	T/T	71
GB56	No amp	wt/wt	−/−	1.76	3.08	0.571	G/T	58
GB57	No amp	wt/wt	−/−	1.16	2.13	0.545	G/T	55
GB63	No amp	wt/wt	−/−	0.75	1.83	0.410	G/G	74
GB8	No amp	wt/wt	−/−	0.67	1.76	0.381	G/T	63
GB84	No amp	wt/wt	−/−	0.57	2.77	0.206	G/T	72
GB94	No amp	wt/wt	−/−	0.91	4.78	0.190	G/T	50
GB41	No amp	wt/wt	−/−	0.28	0.75	0.373	G/T	70
GB9	No amp	wt/wt	−/−	1.19	2.47	0.482	T/T	68
GB1	No amp	wt/wt	−/−	NA	NA	NA	NA	NA
GB51	No amp	wt/−	−/−	NA	NA	NA	T/T	70
GB46	No amp	wt/mut	wt/wt	0.07	0.36	0.194	T/T	46
GB59	No amp	wt/mut	wt/wt	1.14	5.03	0.227	T/T	51
GB221	No amp	wt/mut	wt/wt	0.10	0.48	0.208	G/T	48
GB193	No amp	wt/mut	wt/−	0.37	1.43	0.259	G/T	56
GB27	No amp	mut/mut	wt/wt	1.61	0.47	3.426	T/T	31
GB164	No amp	mut/−	wt/wt	0.29	1.96	0.148	G/T	63
GB61	No amp	mut/−	wt/wt	3.00	1.45	2.069	T/T	68
GB103	No amp	mut/−	wt/wt	4.84	1.32	3.667	G/T	72
GB131	No amp	mut/−	wt/wt	1.37	3.63	0.377	T/T	68
GB29	No amp	mut/−	wt/−	0.09	0.93	0.097	T/T	45
GB132	No amp	mut/−	wt/−	1.51	2.5	0.604	G/T	52
GB17	No amp	wt/mut	−/−	0.86	0.61	1.410	G/T	62
GB138	No amp	wt/mut	−/−	0.22	0.36	0.611	G/G	55
GB22	No amp	wt/mut	−/−	0.31	0.71	0.437	T/T	43
GB5	No amp	wt/mut	−/−	1.07	1.29	0.829	T/T	56
GB4	No amp	wt/mut	−/−	NA	NA	NA	NA	NA
GB166	No amp	mut/−	−/−	0.31	0.5	0.620	T/T	68
GB16	No amp	mut/−	−/−	0.14	0.12	1.167	G/T	73
GB33	No amp	mut/−	−/−	0.52	0.41	1.268	G/G	66
GB55	No amp	mut/−	−/−	1.42	2.01	0.706	T/T	74
GB217X4^b^	Amp	wt/wt/wt	wt/wt	28.8	2.58	11.162	NA	NA
GB181X13^b^	No amp	wt/wt	−/−	NA	NA	NA	T/T	NA
GB166X1^b^	No amp	mut/−	−/−	0.03	0.44	0.068	NA	NA
CCF-STTG1^c^	Amp	wt/wt	wt/−	NA	NA	NA	NA	NA
Tp365MG^c^	Amp	wt/wt	wt/−	NA	NA	NA	NA	NA
Tp265MG^c^	No amp	−/−	−/−	0.41	0.01	41	G/G	NA

A=astrocytoma; AA=anaplastic astrocytoma; GB=glioblastoma; no amp=no amplification; amp=amplification (⩾ 5 copies); wt/wt=two wild-type copies; −/−=homozygous deletion (see text); wt/−=loss of one allele; wt/mut=retention of one wild-type allele and one mutated allele; mut/mut=both alleles with unique mutations; mut/−=loss of one allele and retained allele mutated; NA=not applicable.

a Normalised target/reference (MDM2/18S) ratio.

b Glioblastoma xenografts hold the same number as the tumour from which they were derived with the suffix X followed by passage number.

c Glioblastoma cell lines.
