# Supplementary material for: p53-independent mechanisms regulate the P2-MDM2 promoter in adult astrocytic tumours
Source: Br J Cancer. 2008 Sep 9;99(7):1144–52. doi: 10.1038/sj.bjc.6604643 (PMC2567066; doi:10.1038/sj.bjc.6604643)
Supplement: Supplementary Tables 1 and 2 [file 6604643x1.doc]

**Supplementary Tables**

**Supplementary Table 1.** Specimen No, diagnosis and usage in study

subsections

| **Specimen No^1^** | **WHO Malignancy Grade^4^** | ***MDM2 Promoter Usage^5^*** | ***P1 & P2 Expression Levels^5^*** | ***SNP309 Analysis^5^*** |
| --- | --- | --- | --- | --- |
|  |  |  |  |  |
| A22 | II | √ | √ | √ |
| A54 | II | - | √ | √ |
| A25 | II | - | √ | √ |
| A50 | II | √ | √ | √ |
| A7 | II | √ | √ | √ |
| A23 | II | √ | √ | √ |
| A30 | II | √ | √ | √ |
| AA104 | III | - | √ | √ |
| AA34 | III | √ | √ | √ |
| AA59 | III | √ | √ | √ |
| AA76 | III | - | √ | √ |
| AA107 | III | - | √ | √ |
| AA110 | III | - | √ | √ |
| AA15 | III | √ | √ | √ |
| AA50 | III | √ | √ | √ |
| AA49 | III | √ | - | - |
| AA90 | III | - | √ | √ |
| GB180 | IV | √ | √ | √ |
| GB217 | IV | √ | √ | √ |
| GB267 | IV | √ | √ | √ |
| GB245 | IV | √ | √ | √ |
| GB246 | IV | √ | √ | √ |
| GB35 | IV | √ | √ | √ |
| GB37 | IV | √ | √ | √ |
| GB90 | IV | √ | √ | √ |
| GB140 | IV | √ | √ | √ |
| GB223 | IV | - | √ | √ |
| GB7 | IV | √ | √ | √ |
| GB81 | IV | √ | √ | √ |
| GB237 | IV | √ | - | √ |
| GB96 | IV | - | √ | √ |
| GB75 | IV | - | √ | √ |
| GB149 | IV | √ | √ | √ |
| GB247 | IV | - | √ | √ |
| GB250 | IV | √ | √ | √ |
| GB30 | IV | - | - | √ |
| GB144 | IV | √ | √ | √ |
| GB24 | IV | √ | √ | √ |
| GB18 | IV | √ | √ | √ |
| GB3 | IV | √ | √ | √ |
| GB32 | IV | √ | √ | √ |
| GB34 | IV | √ | √ | √ |
| GB52 | IV | √ | √ | √ |
| GB56 | IV | √ | √ | √ |
| GB57 | IV | √ | √ | √ |
| GB63 | IV | √ | √ | √ |
| GB8 | IV | √ | √ | √ |
| GB84 | IV | √ | √ | √ |
| GB94 | IV | √ | √ | √ |
| GB41 | IV | √ | √ | √ |
| GB9 | IV | √ | √ | √ |
| GB1 | IV | √ | - | - |
| GB51 | IV | - | - | √ |
| GB46 | IV | √ | √ | √ |
| GB59 | IV | √ | √ | √ |
| GB221 | IV | - | √ | √ |
| GB193 | IV | - | √ | √ |
| GB27 | IV | √ | √ | √ |
| GB164 | IV | √ | √ | √ |
| GB61 | IV | √ | √ | √ |
| GB103 | IV | √ | √ | √ |
| GB131 | IV | √ | √ | √ |
| GB29 | IV | - | √ | √ |
| GB132 | IV | √ | √ | √ |
| GB17 | IV | √ | √ | √ |
| GB138 | IV | - | √ | √ |
| GB22 | IV | √ | √ | √ |
| GB5 | IV | √ | √ | √ |
| GB4 | IV | √ | - | - |
| GB166 | IV | √ | √ | √ |
| GB16 | IV | - | √ | √ |
| GB33 | IV | √ | √ | √ |
| GB55 | IV | √ | √ | √ |
| GB217X4^2^ | IV | √ | √ | - |
| GB181X13^2^ | IV | √ | - | √ |
| GB166X1^2^ | IV | - | √ | - |
| CCF-STTG1^3^ | IV | √ | - | - |
| Tp365MG^3^ | IV | √ | - | - |
| Tp265MG^3^ | IV | √ | √ | √ |

^1^A, astrocytoma; AA, anaplastic astrocytoma; GB, glioblastoma

^2^Glioblastoma xenografts hold the same number as the tumour from which they were derived with the suffix X followed by passage number

^3^Glioblastoma cell lines

^4^WHO, World Health Organisation. For details of criteria for malignancy grade, see (Loui*s et* al, 2007)

^5^Indicates case used in the part of the study (**√**, case available for study; -, case not available for study)

**Supplementary Table 2.** Sequences of the primers used

| **Gene** | **Exons** | **Primer** | **Direction** | **Primer sequence** |
| --- | --- | --- | --- | --- |
| *TP53* | Exon 2 | PC1046 | Forward | TCCCCACTTTTCCTCTTGCAG |
| *TP53* | Exon 2 | PC1047 | Reverse | TTTTCGCTTCCCACAGGTCTC |
| *TP53* | Exon 5 | PC929 | Forward | GCCGTGTTCCAGTTGCTTTATC |
| *TP53* | Exon 5 | PC931 | Reverse | GTCGTCTCTCCAGCCCCAGC |
| *DEPDC5* | Exon 35 | PC2419 | Forward | CATCTTTCCTTCCACTTGTTGCC |
| *DEPDC5* | Exon 35 | PC2420 | Reverse | TCGGGACCTAAGCAAACAGCTC |
| *TP53* | Exon 4 | PC180 | Forward | TGGTTCACTGAAGACCCAGGTC |
| *TP53* | Exon 9 | PC50 | Reverse | GGAATTCTCCATCCAGTGGTTTC |
| *TP53* | Exon 7 | PC446 | Forward | CCATCCTCACCATCATCACAC |
| *MDM2* | Exon 1 | PC3176 | Forward | TTTCGCAGCCAGGAGCACCGT |
| *MDM2* | Exon 2 | PC3600 | Forward | CTTTTTCTCTGCTGATCCAG |
| *MDM2* | Exon 3 | PC3291 | Reverse | GGGTCTCTTGTTCCG |
| *MDM2* | Exon 12 | PC3238 | Reverse | CTATGTGAATTGAGGCATTT |
| *MDM2* | Exon 2 | PC4570 | Forward | CTTTTTCTCTGCTGATCCAGGC |
| *MDM2* | Exon 3 | PC4573 | Reverse | CAGGGTCTCTTGTTCCGAAGCTG |

**Supplementary figure legends**

**Supplementary Figure 1.** Agarose gel electrophoresis of RT-PCR products showing expression of P1- and P2-MDM2 transcripts in astrocytic gliomas with different *MDM2*, *TP53* and *p14^ARF^* gene status. The forward primer was located in exon 1 sequence (PC3176) or exon 2 sequence (PC3600) of the MDM2 cDNA. The reverse primer was located in exon 3 sequence (PC3291) of the MDM2 cDNA. The *TP53* allelic status is indicated above each individual case in section B. Note that this is only a preliminary, non-quantitative approach aiming to investigate whether both transcripts with their unique 5’ UTRs could be found in the tumours, xenografts and cell lines. For details of the *MDM2*, *TP53* and *p14^ARF^* gene status of each of the specimens, see Table 1. (A) *MDM2* amp, at least one wt *TP53* allele*,* at least one wt *p14^ARF^*. (B) no *MDM2* amp, variable *TP53* and *p14^ARF^* gene status. (C) no *MDM2* amp, two wt *TP53* alleles, -/- *p14^ARF^*. (D) no *MDM2* amp, two wt *TP53 and p14^ARF^* alleles. Amp, amplification; wt, wild type; mut, mutation; -, loss.
