# Supplementary material for: p53-independent mechanisms regulate the P2-MDM2 promoter in adult astrocytic tumours
Source: Br J Cancer. 2008 Sep 9;99(7):1144–52. doi: 10.1038/sj.bjc.6604643 (PMC2567066; doi:10.1038/sj.bjc.6604643)
Supplement: Supplementary Figure 1 [file 6604643x2.ppt]

## Slide 1
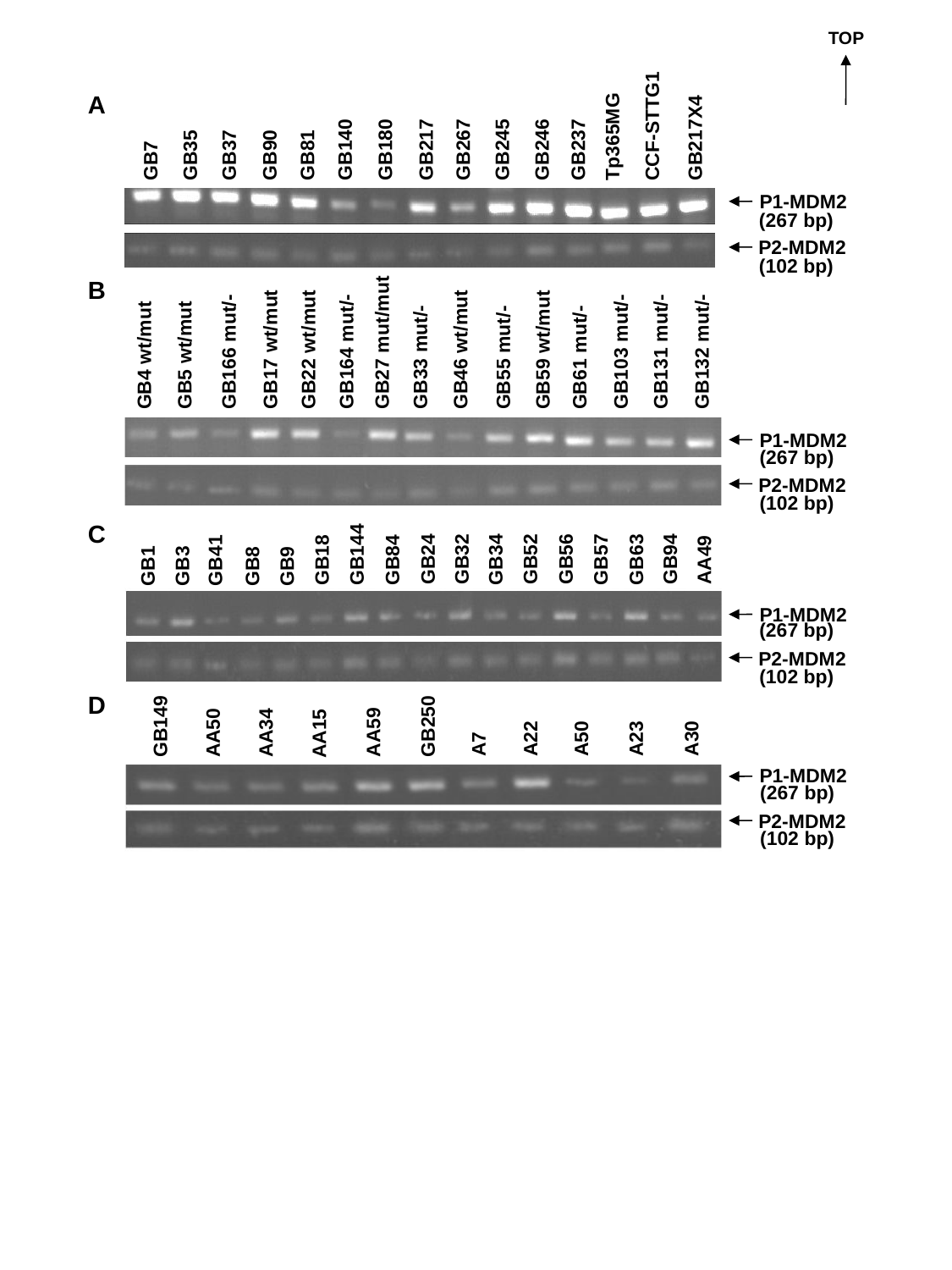

TOP
A
CCF-STTG1
Tp365MG
GB217X4
GB246
GB237
GB90
GB37
GB81
GB140
GB180
GB217
GB267
GB245
GB7
GB35
P1-MDM2
(267 bp)
P2-MDM2
(102 bp)
B
GB5 wt/mut
GB4 wt/mut
GB55 mut/-
GB22 wt/mut
GB17 wt/mut
GB27 mut/mut
GB103 mut/-
GB132 mut/-
GB59 wt/mut
GB131 mut/-
GB46 wt/mut
GB61 mut/-
GB164 mut/-
GB166 mut/-
GB33 mut/-
P1-MDM2
(267 bp)
P2-MDM2
(102 bp)
GB24
GB56
GB34
GB84
GB32
GB52
GB144
GB3
GB41
GB8
GB9
GB94
AA49
GB57
GB63
C
GB18
GB1
P1-MDM2
(267 bp)
P2-MDM2
(102 bp)
A30
A22
GB149
A50
A23
A7
GB250
AA34
AA15
D
AA59
AA50
P1-MDM2
(267 bp)
P2-MDM2
(102 bp)
